# Probing hemoglobin glyco-products by fluorescence spectroscopy

**DOI:** 10.1039/c9ra05243g

**Published:** 2019-11-19

**Authors:** Aristos Ioannou, Constantinos Varotsis

**Affiliations:** Cyprus University of Technology, Department of Environmental Science and Technology Limassol Cyprus c.varotsis@cut.ac.cy +357 25002802

## Abstract

Maillard reaction products (MRPs) participate in reactions of carbohydrate intermediates with proteins, resulting in the formation of advanced glycation end-products (AGEs). Dietary Maillard reaction products are recognized as potential chemical modifiers of human proteins. We have investigated the reaction of isolated MRPs from an asparagine–glucose model system with hemoglobin (Hb) to elucidate the binding effect of the MRPs in hemoglobin by fluorescence spectrophotometry. The tryptophan-specific fluorescence obtained for glycated hemoglobin exhibited a Stokes effect since the wavelength of the emission peak was shifted to a higher wavelength than that of native Hb. The formation of new fluorescence emission features indicates the formation of modified hemoglobin species. Fluorescence spectroscopic studies provide evidence that the conformational changes in the β-Trp 37 moiety induce motion of the distal His 64 (E7) in the heme binding pocket. This results in the formation of inactive hemichrome forms of hemoglobin which are related to blood disorders.

## Abstract 2

Fluorescence mapping of hemoglobin AGE formation after hemoglobin modification by Maillard reaction products.

## Graphical abstract

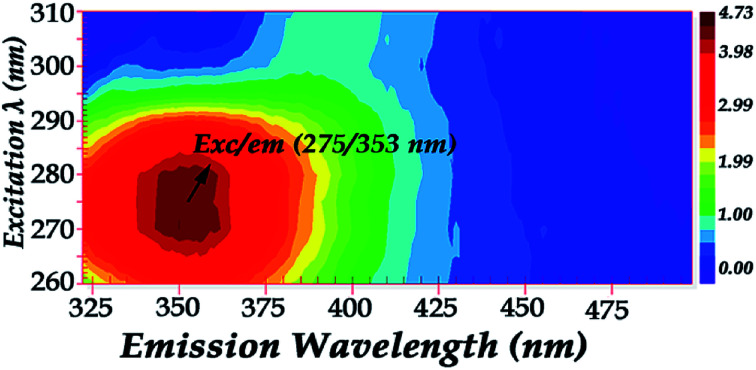

## 1. Introduction

Maillard Reaction Products (MRPs) are attributes of thermally processed foods, contributing to their flavor, aroma and texture.^1–4^ The increased consumption of MRPs in recent years has pointed towards their implication in the development of processes such as diabetes and degenerative diseases. Advanced glycation end products (AGEs) lead to the formation of unstable, reactive intermediates that readily form intra- and intermolecular covalent crosslinks or glyco-oxidation products involved in the vascular and renal complications of diabetes mellitus.^5^ People with diabetes and renal disease display elevated serum AGE levels and reduced urinary AGE excretion. Unreactive AGEs can readily form new crosslinks with low density lipoprotein (LDL) or collagen. It has been demonstrated that AGEs absorbed into the blood stream may represent a major source of chemically and biologically active toxins. These glycotoxins are only partially eliminated in the urine and may exert significant reactivity in the body. Persons with impaired renal functions are unable to efficiency excrete glycotoxins in the urine, resulting in abnormally high AGE concentrations in the blood and tissues. Hemoglobin is the most abundant blood protein and consists of two α and β subunits which are non-covalently associated within erythrocytes as a 64.5 kDa tetramer.^5^ The natural mechanisms of Hb uptake and decomposition, the latter of which leads to unloading of any bound compounds, can be employed to selectively monitor the Hb-bound complexes.

Recently, the isolation of reaction products of asparagine with reducing sugars at high pH and temperature was explored by high performance liquid chromatography (HPLC) coupling methods.^6–8^ Spectroscopic analysis of selected Maillard reaction products demonstrated the appearance of asparagine–saccharide conjugates.^7^ These were shown to induce the formation of a hemichrome species in hemoglobin, which obstructs the ligand binding site irreversibly.^8^ The intrinsic fluorescence of tryptophans (W) and tyrosines (Y) in Hb has been used as an important probe in understanding the dynamics and conformational changes induced by ligand binding and to elucidate the binding location and relevant parameters related to binding interactions.^9–11^ Excitation with light at 280 nm reflects contribution from both W and Y residues whereas excitation at 296 nm is used to distinguish the contribution of W residues alone without any fluorescence energy transfer from the Y residues. The assignment of β37-Trp as the origin of the emitted fluorescence in Hb when excited by light at 296 nm has been established providing solid evidence that this spectral feature is sensitive to conformational changes in Hb.^9–11^ Anomalous emissions upon excitation at 280 nm have been attributed to a combination of tyrosine and tyrosinate emissions. Tryptophan fluorescence measurements provide a reliable analytical tool to characterize the tryptophan environment which is related to the tertiary conformation. A rigid environment around the Trp and a change in polarity affects the emission of the Trp fluorescence spectrum. A blue shift in the fluorescence emission indicates the presence of a hydrophobic environment around the Trp and increased fluorescence intensity indicates a more rigid environment.^9,10^ The fluorescence of advanced Maillard reaction products has also been investigated and the potential application in food sciences has been reported.^12–15^

In this study, we decided to use fractions of an asparagine/carbohydrate system to glycate hemoglobin in order to demonstrate that other glycated species may contribute towards the pool of glycated proteins in the blood. The reaction of the discrete MRPs originated from an asparagine–glucose model system with hemoglobin was investigated after one day and one month incubation time by fluorescence excitation/emission spectrophotometry. These specific time points were selected to demonstrate short and long-term exposure, respectively, of hemoglobin to these glycated species. The Trp-specific fluorescence obtained for the glycated-Hb complexes exhibited Stokes effect which is attributed to the formation of Hb–MRPs complexes and to structural modification of Hb induced by the MRPs. We propose that the MRPs interact through hydrophobic and hydrogen bonding in the moiety of β-37 Trp, and the interaction perturbs the structural and functional properties of Hb. We suggest that the conformational changes at the N1H site have a direct effect in the O_2_ binding site forming a hemichrome, and thus, β-37 Trp may be useful as an intrinsic probe to study the dynamic processes involved in the formation of hemichromes which are related to blood disorders.^5,8^ Furthermore, comparison of the fluorescence data in the presence and absence of the MRPs with UV resonance Raman data indicate that the microenvironment of β37Trp which includes αTyr140 which is located in the microenvironment of β37Trp, α43Tyr, and β145Tyr is involved in the R → T transition.^8^

## 2. Experimental

### 2.1 Sample preparation of MRPs

Solutions of glucose and asparagine (0.2 M, pH 8.0) were prepared in phosphate buffer (50 mM, pH 8.0). Samples were heated in closed screw-capped tubes at 180 °C in a heating oven (Memmert, Germany) for 2 hours.

### 2.2 HPLC-fraction collector analysis of MRPs

The HPLC instrumental setup and the separation of individual MRP fractions were described in detail earlier.^7,8^ The HPLC experimental setup consisted of a Varian 218 Prepstar Solvent Delivery Module, an Agilent Manual FL-Injection Valve, an Agilent 1260 Infinity Variable Wavelength Detector (VWD) and an Agilent 440 LC Fraction Collector. HPLC analysis was performed on a 4.6 × 250 mm, 5 μm particle size, Zorbax SB-Aq analytical column (Agilent Technologies). Maillard reaction products (MPRs) were analyzed under aqueous conditions using a previously reported method.^7^ Water at a flow rate of 0.5 ml min^−1^ was used isocratically as the mobile phase at room temperature. Maillard reaction products (MPRs) were detected at 200 nm.

### 2.3 HPLC-fraction collector analysis of MRPs

Hemoglobin from bovine blood (Sigma) at approximately 1 mM concentration in 50 mM phosphate buffer (pH 8.0) was diluted hundred-fold to avoid inner filter effects during fluorescence measurement. The diluted hemoglobin was immediately mixed with each individual LC fraction (2 : 1 v/v) from the reaction between asparagine and glucose at 180 °C. The solutions were mixed and kept at room temperature. The fluorescence spectra of the mixtures were collected up to 30 days incubation.

### 2.4 Fluorescence analysis

The Cary Eclipse Fluorescence Spectrophotometer (Agilent Technologies, USA) controlled from a PC using the Agilent Technologies WinFLR software (version 1.2) was used for obtaining the fluorescent signal. Fluorescence values of the native and glycated hemoglobin samples were measured using a clear four-sided quartz cuvette in the spectrophotometer sample compartment. The excitation and emission slit widths for both the excitation and emission monochromators were set with the value of 5.0 nm. The Excitation–Emission Matrix (EEM) measurement and processing were generated by scanning emission spectra from 320 nm to 500 nm at 1 nm intervals, with 5 nm increments of the excitation wavelengths from 260 nm to 310 nm. The Milli-Q water blank was subtracted from the sample EEM spectra.

## 3. Results and discussion

Maillard reactions are highly influenced by pH and temperature.^4^ Model systems similar to reactions naturally occurring in processed foods that have undergone high temperature treatments are useful in resembling more complex chemical systems occurring in foods. Maillard reaction rate is highly increased by the combination of factors such as slightly alkaline pH and high temperatures.^7,8^ In this context, we have chosen pH 8 and a temperature of 180 °C in order to produce these Maillard reaction intermediates in sufficient concentrations for subsequent isolation. Our choice of pH aimed to keep a balance between our model Maillard system and the physiological pH of hemoglobin in human blood which is slightly alkaline.

**Fig. 1:**
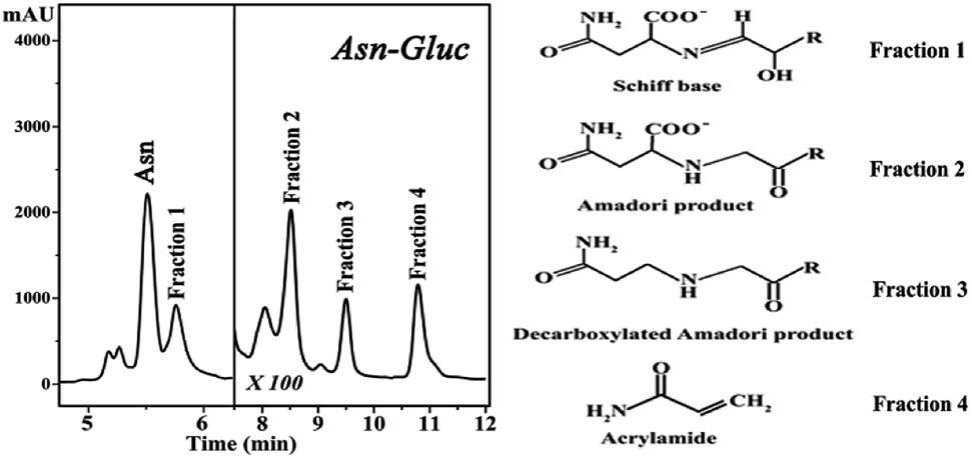
High performance liquid chromatography (HPLC) chromatograms of the reaction mixture of asparagine and glucose (pH 8.0, 180 °C).

The HPLC chromatograms of the asparagine–glucose reactions are shown in Fig. 1. A major signal at Rt = 5.7 min is present which corresponds to the excess unreacted asparagine and other smaller chromatographic peaks arising from individual MRPs. Fraction 1 corresponds to the Schiff base and Fractions 2, 3 and 4 have been characterized to originate from the Amadori product, decarboxylated Amadori product and acrylamide, respectively.^7^ These fractions were incubated with Hb in a one day and one month period and their effect on hemoglobin was probed by fluorescence spectroscopy.

Proteins react with glucose to form a stable Amadori product through Schiff base adducts that are transformed to advanced glycation end products (AGEs) some of which exhibit fluorescence.^14–16^ Therefore, their fluorescence intensities indicate of the extent of AGE-modified proteins. These excitation–emission maxima are distinct from tryptophan fluorescence in proteins. A worthy feature of intrinsic protein fluorescence in Hb is the high sensitivity of the local environment of β-37 Trp.^9–11^ Alterations in the emission spectra of tryptophan may occur in response to conformational transitions, subunit association, substrate binding, and/or denaturation.

**Fig. 2:**
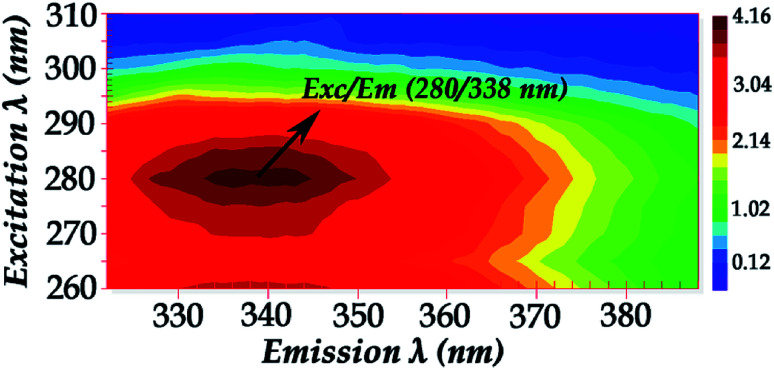
Fluorescence excitation–emission matrix (EEM) of native hemoglobin (pH 8.0) (excitation wavelength range: 260–310 nm).

Fig. 2 shows the fluorescence excitation–emission matrix (EEM) of native hemoglobin (pH 8.0). Hb displays weak fluorescence under these conditions when maximally excited at 280 nm. The contour map enables the identification of peaks within a multi-component sample matrix. The different colors are associated with intensity with the deep red being the most intense. It is evident that for native hemoglobin the maximum excitation/emission is 280 nm/338 nm (Fig. 2). Excitation at 280 nm will excite both tyrosine and tryptophan residues. The signals from the 280 nm excitation are three times more intense and thus small changes can be determined more reliably. The fluorescence spectra shown in Fig. 2 are in good agreement with those previously reported.^9,10^ Upon excitation at 296 nm, the Trp residues are selectively excited and the majority of the fluorescence observed in Hb has been previously assigned to the β-37 Trp residue. The observed difference in fluorescence intensity between the T and R states in Hb has been attributed to the changes occurring at β-37 Trp. Therefore, the Trp-37 in the β-chains of Hb is the fluorophoric moiety of Hb and can serve as the marker residue for monitoring the dynamics of Hb-complexes. It is known that α-140Tyr which is located in the microenvironment of β-37 Trp, β-42 Tyr, and β-145 Tyr undergo conformational changes during the R → T transition. A dramatic change in fluorescence intensity could result from altered Tyr → Trp and Trp → heme energy-transfer pathways.^9,10^

**Fig. 3:**
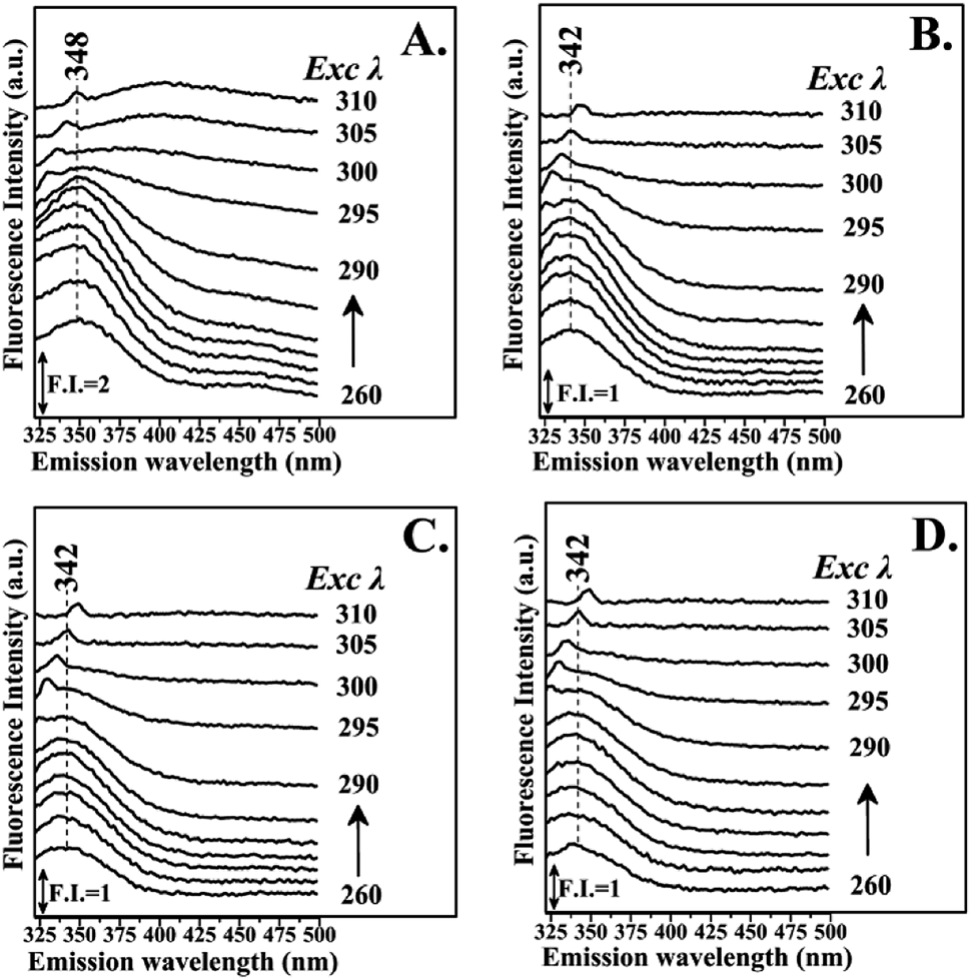
Fluorescence emission spectra of hemoglobin with: Panel A: LC Fraction 1, Panel B: LC Fraction 2, Panel C: LC Fraction 3, Panel D: LC Fraction 4 from the reaction of Asn–Gluc after 1 day incubation (excitation wavelength range: 260–310 nm).

**Fig. 4:**
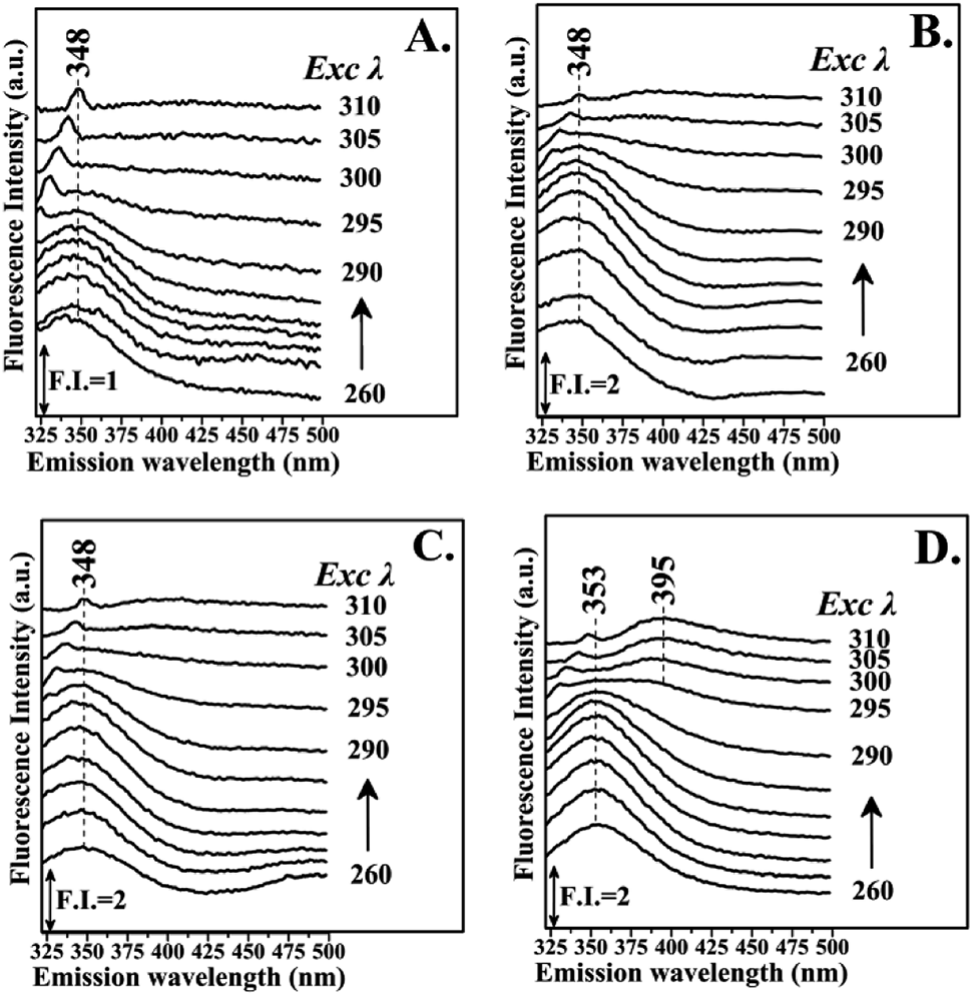
Fluorescence emission spectra of hemoglobin with: PanelA: LC Fraction 1, Panel B: LC Fraction 2, Panel C: LC Fraction 3, Panel D: LC Fraction 4 from the reaction of Asn–Gluc after 1 month incubation (excitation wavelength range: 260–310 nm).

Fig. 3 shows the fluorescence emission spectra of the reactions of Hb with the Schiff base (Fraction 1), Amadori product (Fraction 2), decarboxylated Amadori product (Fraction 3) and acrylamide (Fraction 4) after 1 day of incubation. The Raman peak of H_2_O which is present in all the fluorescence spectra is always located of 3400–3600 cm^−1^ lower in energy than the excitation wavelength. In the 260–295 nm excitation range, the emission maxima were located at 348 nm (Fig. 3A) for the Schiff base (Fraction 1) and approximately at 342 nm (Fig. 3B–D) for the Amadori product (Fraction 2), decarboxylated Amadori product (Fraction 3) and acrylamide (Fraction 4). The fluorescence obtained for these glycated Hb complexes exhibited a Stokes effect since the wavelength of the emission peak is shifted 10 nm in the Hb–Schiff base complex and 4 nm in the Hb–Amadori complex, Hb–decarboxylated Amadori product and Hb–acrylamide complexes. The lowering of this energy level can be associated to conformational changes of Hb. From our experimental results after 1 day incubation it was evident that all 4 fractions achieve the initial binding to hemoglobin as there is a conformational change in the hemoglobin molecule as depicted by the fluorescence spectral shifts. The origin of the fluorescence when excited by light at 280 nm is attributed to tyrosine and tryptophan emissions. The decreased intensity that accompanied the emission shift serves as evidence that this conformational change arises from β-37 Trp at the α_1_β_2_ interface when the Hb-complexes are excited by light at 296 nm.^10^ No emissions are observed in the 300–310 nm excitation range. It is generally accepted that when the tryptophan residue becomes hydrogen bonded or increasingly exposed to water, its fluorescent emission shifts to longer wavelengths. Therefore, the increased hydrophilicity can arise as a result of a conformational change coupled to increased exposure of β-37 Trp to the solvent. The observed behavior of the Hb complexes with respect to the contribution of the β-37 Trp environment at the α_1_β_2_ interface is similar to that observed during the R → T transition.^9,10^Fig. 4 illustrates the fluorescent spectra of the hemoglobin–MRP mixtures after 1 month incubation. There is a noticeable shift to longer wavelengths in the emission spectra of the Hb–Amadori complex, Hb–decarboxylated Amadori product and Hb–acrylamide complexes as compared to those spectra observed after one day of incubation, shown in Fig. 3B–D, whereas that of the Hb–Schiff base complex does not show any further change in fluorescent characteristics as that observed in the spectra in Fig. 3A after one day of incubation. This may denote that there was no significant Hb–AGE formation in this case. On the contrary, for the other 3 fractions there are further fluorescence shifts after this time frame, demonstrating further structural change in hemoglobin and Hb–AGE formation. The 6 nm shift of the Hb–Amadori complex and Hb–decarboxylated Amadori product indicates that prolonged incubation times induce a further conformational change at the β-37 Trp microenvironment and the 11 nm shift of the Hb–acrylamide complex indicates strong H-bonded interactions and conformational changes in the moiety of the β-37 Trp residue. The appearance of a new strong emission feature around 395 nm at the excitation range of 295–310 nm is attributed to fluorescence quenching effect on tryptophan fluorescence. This effect was often utilized to demonstrate ligand–protein interactions.^16^ A similar 390 nm emission band was recently identified from fluorescent intensity maps of degenerated cartilage from osteoarthritis patients. The authors discussed that this was a result of non-enzymatic glycation reaction which is followed by the formation of several types of fluorescent crosslinks (AGEs).^17^ Hb–AGE fluorescent crosslinks are likely to result in loss of protein functionality as it is evident from the Hb–acrylamide fluorescent profile.

**Fig. 5:**
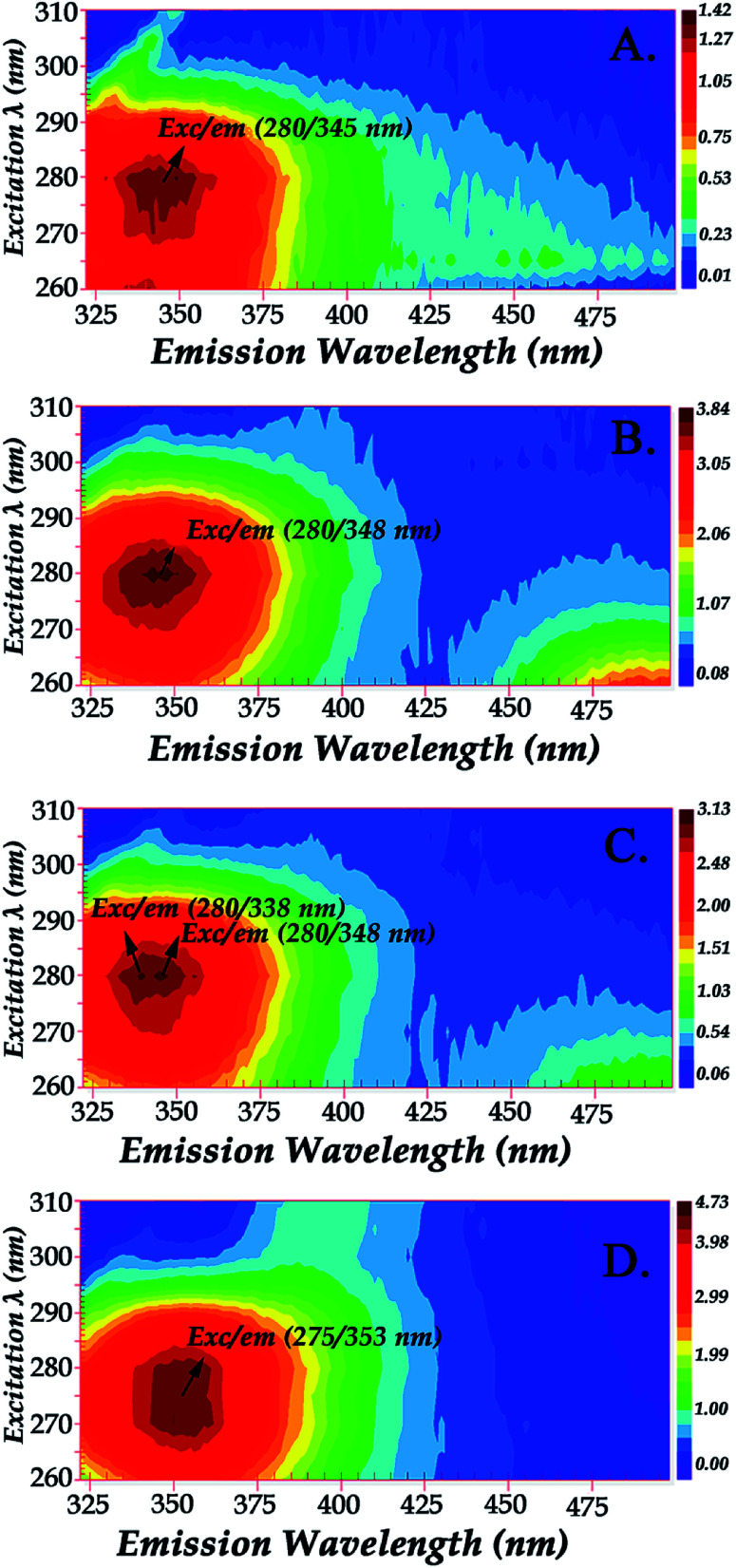
Fluorescence mapping of hemoglobin with: Panel A: LC Fraction 1, Panel B: LC Fraction 2, Panel C: LC Fraction 3, Panel D: LC Fraction 4 from the reaction of Asn–Gluc after 1 month incubation (excitation wavelength range: 260–310 nm).

Glycation process results in two different products: early and advanced glycation endproducts (AGEs). Hb–AGEs formation is a slower process and occurs after prolonged exposure to glycated species. These events are more predominant after 1 month incubation based on their relative intensities as illustrated by fluorescence mapping of incubated hemoglobin in Fig. 5. In Fig. 5 the fluorescence mapping after one month of incubation of hemoglobin with the LC fractions 1–4 are presented. The fluorescent chemical mapping analysis when compared with the corresponding analysis of Hb shown in Fig. 2 demonstrates the effect of MRPs in the moiety of β-37 Trp residue and shows that all the Hb–MRPs complexes are highly heterogeneous systems.

Shifts in fluorescence emission and fluorescence quenching effects are indicative of protein modification in this relatively simple model system. Hb contains three tryptophan residues, but only β-37 Trp is located at the interface between the two dimers. The β-37 Trp residues are located in helices C of the β-subunits and are involved in contacts with the segments FG of the α-subunits at the interdimeric α_1_β_2_ and α_2_β_1_ interfaces of the hemoglobin tetramer.^18^ The intrinsic fluorescence signal of Hb arises from the indole group of tryptophan β-37 and has been used to determine the changes in the quaternary structure of Hb upon ligand binding.^19^ In addition, the energetic changes that occur on ligand binding in human hemoglobin are determined by measurements of the exchange rates of the indole proton of β-37Trp (C3) and the increased exchange rates of the indole proton of β-37Trp determine ligand binding. The interface regions experience a change in structural free energy upon ligand binding and these hinge regions are involved in the transmission of free energy during its allosteric transition.^19^

Results that involve Trp-37 were also obtained to establish the effectiveness of natural drug delivery mechanisms and investigate the interaction between the drug and it natural carrier Hb. It is thus well established that β-37 Trp serves as good optical probe for monitoring ligand binding far away (∼15 Å) from the oxygen binding site.^11^

Hemichromes are formed when hemoglobin undergoes conformational changes resulting in the formation of a six-coordinated Fe^3+^ low-spin His–Fe–His species.^20–22^ Hemoglobin A (HbA) in humans can form hemichromes even under physiological conditions as a result of pH and temperature alterations, and in the autoxidation of oxyHb.^20^ We have recently demonstrated that tetrameric Hbs form a partial hemichrome state upon addition of MRPs compounds. The results presented here in conjunction with our previous observations strongly indicate that Trp-37 plays a critical role in the formation of the hemichrome.^8,20–22^ Obviously, the Maillard reaction species act as external ligands to a protein site near the β-37 Trp moiety, triggering the formation of six-coordinated bis-histidyl hemichrome species.^23,24^ The coordination of the distal His 64 (E7) to the iron produces a scissoring motion in helices E and F which results in modifications in the tertiary structure of the tetramer, mainly in the αβ interface which is critical in the transition from the R to the T state.^23^ Modification of the EF fragment exposes the heme to a more solvent-exposed position narrowing the heme pocket as the E- and F-helices move toward each other.^8,23,24^

## 4. Conclusions

Maillard reaction products (MRPs) which are absorbed in the blood stream represent a major source of chemically and biologically active toxins. These dietary glycotoxins in high local concentration and time bind irreversibly to Hb. Intrinsic fluorescence activity accompanied by fluorescent mapping was employed in this study to provide experimental evidence regarding the relationship of intrinsic Hb fluorescence and the chemical events that follow interactions with glycotoxins originated form the Maillard reaction products. The data presented here demonstrate that the primary interactions of the MRPs with β-37 Trp forming the initial Hb–MRPs complexes are followed by major modifications under prolonged period of time forming the final Hb–MRPs. The present data and those previously reported suggest that β-37 Trp plays a major role in forming the inactive Hb hemichrome complexes, as the result of the presence of the Maillard reaction products near the β-37 Trp moiety of Hb. The implication of the non-enzymatic glycation reaction is the formation of fluorescent cross linked species (AGEs) as a result of the inactive hemichrome form of hemoglobin.^25^ The small number of Maillard reactions being addressed in model reactions in this investigation may point towards the need of a larger *in vivo* study that will extend the applicability of this fluorescence technique in combination with structure sensitive techniques such as FTIR and Raman spectroscopies to a more heterogeneous *in vivo* system.^26–30^

## Conflicts of interest

There are no conflicts to declare.

## Supplementary Material
